# Comparison of general maternal and neonatal conditions and clinical outcomes between embryo transfer and natural conception

**DOI:** 10.1186/s12884-020-03066-9

**Published:** 2020-07-27

**Authors:** Haiyan Pan, Xingshan Zhang, Jiawei Rao, Bing Lin, Jie Yun He, Xingjie Wang, Fengqiong Han, Jinfeng Zhang

**Affiliations:** 1 School of Public Health, Guangdong Medical University, Dongguan, 523808 Guangdong Province China; 2 Shunde Women and Children’s Hospital of Guangdong Medical University, Shunde, 528399 Guangdong Province China

**Keywords:** Embryo transfer, Natural conception, Clinical outcome, Assisted reproductive technology

## Abstract

**Background:**

To examine the differences between pregnant women who underwent embryo transfer (ET) and those who conceived naturally, as well as differences in their respective babies, and to determine the causes for these differences, to provide recommendations for women who are planning to undergo ET.

**Methods:**

A retrospective cohort study was performed of women who had received ET and those who had natural conception (NC) who received medical services during pregnancy and had their babies delivered at the Shunde Women and Children’s Hospital of Guangdong Medical University, China between January 2016 and December 2018. In line with the requirements of the ethics committee, before the formal investigation, we first explained the content of the informed consent of the patient to the patient, and all the subjects included agreed to the content of the informed consent of the patient. Respondents agreed to visit and analyze their medical records under reasonable conditions. Each case in an ET group of 321 women was randomly matched with three cases of NC (963 cases) who delivered on the same day. The demographic information, past history, pregnancy and delivery history, and maternal and neonatal outcomes of the two groups were compared using univariate analysis.

**Results:**

Age, duration of hospitalization, number of pregnancies, number of miscarriages, induced abortion, ectopic pregnancy, gestational diabetes mellitus, preeclampsia, gestational anemia, pregnancy risk, mode of fetal delivery, and number of births were significantly different between the two groups (all *P* < 0.05). However, there were no significant differences in the disease, allergy, infection and blood transfusion histories of the pregnant women, or differences in prevalence of gestational hypothyroidism, gestational respiratory infection, premature rupture of membrane, placental abruption, fetal death, stillbirth, amniotic fluid volume and amniotic fluid clarity between the two groups (all *P* > 0.05). The percentages for low birth weight and premature birth were significantly higher in the ET group than in the NC group. In contrast, infant gender and prevalence of fetal macrosomia, fetal anomaly, neonatal asphyxia, and extremely low birth weight were not significantly different between the two groups (all *P* > 0.05).

**Conclusions:**

The clinical outcomes of mothers and the birth status of infants were better in the NC group than in the ET group. Maternal health must be closely monitored and improved in the ET group to reduce the incidence of gestational comorbidity and enhance the quality of fetal life.

## Background

Increasingly, couples are turning to assisted reproductive technology (ART) for help with conceiving and ultimately giving birth to a healthy live baby of their own [1]. In recent years, there has been increasing concern regarding the safety of ART, due to the potential health impact on these infants. At present, multiple studies have suggested that in vitro fertilization (IVF) pregnancies may be at increased risk for preterm birth, low birth weight, congenital anomalies, perinatal mortality and several other pregnancy-related complications compared to unassisted pregnancies [2]. Concerns have been raised over an increased risk of adverse maternal outcomes like gestational diabetes mellitus and preeclampsia in ART populations as compared with the natural conception group [3].

In this study, we conducted a questionnaire survey of eligible subjects to determine the differences in clinical outcomes of mothers and their newborns between embryo transfer (ET) and natural conception (NC) and to provide data information for the embryo transfer information database of Shunde and reference information for the mother who is about to undergo embryo transfer.

## Methods

### Subjects

The subjects for this study were 321 women who had ET and their babies and 963 women who conceived naturally (natural conception, NC) and their babies, who delivered in the Shunde Women and Children’s Hospital of Guangdong Medical University between January 2016 and December 2018.

## Method

The general information of the 321 women who underwent ET in the hospital and their babies was subjected to a retrospective analysis. Each ET case was randomly matched with three NC cases born on the same day as the ET case, and a total of 963 NC cases were used as controls. Simple random sampling was used. We determine the time of delivery for the mother of the embryo transfer and then look for the mother of the naturally born fetus on the same day. These mothers included both vaginal and cesarean deliveries. We number the mothers who gave birth naturally on the same day, and randomly select the corresponding mothers through computer software. This proportion could achieve satisfactory research results and the workload was more appropriate. The general conditions and clinical outcomes of the women during their pregnancy and their babies were compared between the ET and NC groups.

### Source of information

Original data from the admission records were transferred to a paper data collection questionnaire form. The same information was retrieved for both groups and included general demographic information, past medical history, history of pregnancy and delivery, and clinical outcomes of the mothers (maternal comorbidities and current delivery records) and infants (gender, premature birth, birth weight, birth defects, and neonatal asphyxia). According to the basic situation and pregnancy complications of each mother during and after pregnancy, the pregnancy status of pregnant women is divided into I, II and Ш categories, from low-risk to high-risk. The first grade is general pregnancy, the second grade is general high-risk pregnancy and the third grade is serious high-risk pregnancy. At the same time, it can meet two or more categories, with the high category as the classification standard. Class II or above is high-risk pregnancy, which means there are certain complications, complications or pathogenic factors in the process of pregnancy, which may cause harm to pregnant women, fetuses and newborns or cause dystocia.

### Statistical analysis

Data were entered using Excel and EpiData 3.1, and were statistically analyzed using SPSS 22.0 (IBM). Categorical data were expressed as frequency (%) and compared using the ***χ***^***2***^ test. Continuous data were expressed as mean ± standard deviation ($\overline{x}$ ±*s*) and normally distributed data were compared using the independent samples *t*-test. When comparing the ET group with the NC group we adjusted for confounding factors (parity, BMI and maternal age) by using logistic (categorical outcomes) regression analyse. *P* < 0.05 was considered statistically significant.

## Results

### Information on ET group and NC group from January 2016 to December 2018

A total of 22,775 babies were born between January 2016 and December 2018 in the Shunde Women and Children’s Hospital of Guangdong Medical University. The total number of pregnant women receiving embryo transfer during this period was 321. The proportion of infants conceived by embryo transfer was 1.4%.

### Demographic information

The results showed that ET group had a significantly higher age compared to NC group, both before and after adjusting for confounding factors. The percentage of women who were ≥ 35 years old while pregnant was higher in the ET group. However, occupation, marital status and educational background had no significant differences between the two groups after adjusting for confounding factors. (See Table 1).

**Table 1:** Comparison of demographic information of pregnant women between the ET and NC groups

Demographic information	ET group, ***n*** (%)	NC group, ***n*** (%)	***χ***^***2***^	***P***	***Adjusted OR***	***95%CI***	***P***
Age			45.549	< 0.0001	1.233	1.176–1.293	< 0.0001
Advanced (≥35 years old)	125 (38.9)	194 (20.1)					
Non-advanced (< 35 years old)	196 (61.1)	769 (79.9)					
Occupation			15.647	0.016	0.984	0.910–1.014	0.687
Enterprise staff	77 (24.0)	193 (20.0)					
Professional/technical personnel	14 (4.4)	19 (2.0)					
Worker	14 (4.4)	64 (6.6)					
Farmer/freelancer	10 (3.1)	53 (5.5)					
Unemployed	139 (43.3)	400 (41.5)					
Seeking employment	11 (3.4)	60 (6.2)					
Other	56 (17.4)	174 (18.1)					
Marital status			9.924	0.007	1.407	0.907–2.181	0.127
Single	2 (0.6)	41 (4.3)					
Married	316 (98.4)	915 (95.0)					
Remarried/remarried to ex- spouse/divorced	3 (0.9)	7 (0.7)					
Educational background			36.382	< 0.001	1.196	0.901–1.587	0.216
Elementary and below	12 (3.7)	42 (4.4)					
Secondary school	138 (43.0)	500 (51.9)					
Post-secondary school	105 (32.7)	341(35.4)					
Bachelor degree and higher	66 (20.6)	80 (8.3)					

*ET* Embryo transfer, *NC* natural conception

### Comparison of medical past history between the embryo transfer and natural conception groups

There were no significant differences in the history of disease, allergy infection and blood transfusion between the two groups (*P* > 0.05). However, the proportion of women with a history of surgery was significantly higher in the ET group (*P* < 0.05) (See Table 2).

**Table 2:** Comparison of past medical history between the ET and NC groups

Past medical history	ET group, ***n*** (%)	NC group, ***n*** (%)	***χ***^***2***^	***P***	***Adjusted OR***	***95%CI***	***P***
Any disease	12 (3.7)	40 (4.2)	0.107	0.744			
Allergy	17 (5.3)	29 (9.0)	3.638	0.056			
Infection	5 (1.6)	3 (0.3)	4.193	0.041	2.226	0.527–2.400	0.276
Surgery	110 (34.3)	143 (14.8)	57.378	< 0.001	3.926	2.661–5.793	< 0.001
Blood transfusion	1 (0.3)	4 (0.4)	0	1			

*ET* Embryo transfer, *NC* natural conception

### Comparison of pregnancy and delivery history between the embryo transfer and natural conception groups

The number of pregnancies, miscarriages, induced abortions and ectopic pregnancies was significantly different between the ET and NC groups (all *P* < 0.05). The percentage of women who had experienced > 1 pregnancy was higher in the NC group. In contrast, ectopic pregnancy had occurred more in the ET group. There were no significant differences between the two groups in the number of spontaneous abortion, fetal deaths and stillbirths (*P* > 0.05; See Table 3).

**Table 3:** Comparison of pregnancy and delivery history between the ET and NC groups

History of pregnancy and delivery	ET group, ***n*** (%)	NC group, ***n*** (%)	***χ***^***2***^	***P***	***Adjusted OR***	***95%CI***	***P***
Number of pregnancies (including any previous and current pregnancy)			30.208	< 0.001	0.082	0.057–0.118	< 0.001
1	154 (48.0)	299 (31.0)					
> 1	167 (52.0)	664 (69.0)					
Number of miscarriages			7.56	0.006	0.736	0.569–0.952	0.02
0	231 (72.0)	575 (59.7)					
≥ 1	108 (33.6)	388 (40.3)					
Spontaneous abortion			5.448	0.02	1.353	0.944–1.939	0.099
Yes	35 (10.9)	66 (6.85)					
No	286 (89.1)	897 (93.1)					
Induced abortion			16.723	< 0.001	0.674	0.529–0.859	< 0.001
Yes	75 (23.4)	344 (35.7)					
No	246 (76.6)	619 (64.3)					
Fetal death			–	0.604			
Yes	2 (0.6)	3 (0.3)					
No	319 (99.4)	960 (99.7)					
Stillbirth			–	1			
Yes	0 (0.0)	2 (0.2)					
No	321 (100.0)	961 (99.8)					
Ectopic pregnancy			92.704	< 0.001	3.131	1.921–5.103	< 0.001
Yes	44 (13.7)	11 (1.1)					
No	277 (86.3)	952 (98.9)					

*ET* Embryonic transfer, *NT* natural conception. – indicates no *χ*^*2*^ value

### Comparison of maternal clinical outcomes between the embryo transfer and natural conception groups

#### Maternal comorbidities

The incidence rates of gestational diabetes mellitus (GDM), gestational anemia and preeclampsia were significantly higher in the ET group than in the NC group (all *P* < 0.05). There were no significant differences in the incidence of other maternal comorbidities between the two groups (*P* > 0.05). The incidence of threatened labor was significantly higher in the NC group (*P* < 0.05; See Table 4).

**Table 4:** Comparison of maternal comorbidities between the ET and NC groups

Maternal comorbidity	ET group, ***n*** (%)	NC group, ***n*** (%)	***χ***^***2***^	***P***	***Adjusted OR***	***95%CI***	***P***
Gestational diabetes mellitis			30.761	< 0.001	1.58	1.151–2.168	0.005
Yes	135 (42.1)	254 (26.4)					
No	186 (58.0)	732 (76.0)					
Gestational hypertension			0	1			
Yes	4 (1.2)	11 (1.1)					
No	317 (98.8)	952 (98.9)					
Gestational anemia			32.894	< 0.001	5.306	2.894–9.927	< 0.001
Yes	40 (12.5)	36 (3.7)					
No	281 (87.5)	927 (96.3)					
Preeclampsia			16.428	< 0.001	3.362	1.308–8.638	0.012
Yes	17 (5.3)	13 (1.3)					
No	304 (94.7)	950 (98.7)					
Gestational hypothyroidism			2.705	0.1			
Yes	8 (2.5)	10 (1.0)					
No	313 (97.5)	953 (99.0)					
Gestational respiratory infection			2.494	0.114			
Yes	9 (2.8)	14 (1.5)					
No	312 (97.2)	949 (98.5)					
Premature rupture of the membrane			0.177	0.674			
Yes	55 (17.1)	175 (18.2)					
No	266 (82.9)	788 (81.8)					
Placental abruption			0.179	0.672			
Yes	3 (0.9)	14 (1.5)					
No	318 (99.1)	949 (98.5)					
Gestational comorbidities			31.858	< 0.001	0.437	0.287–0.665	< 0.001
Yes	211 (65.7)	458 (47.6)					
No	110 (34.3)	505 (52.4)					
Reasons for miscarriage prevention treatment			42.122	< 0.001	0.075	0.688–0.806	< 0.001
Threatened labor	82 (25.5)	551 (57.2)					
Threated premature labor	24 (7.5)	41 (4.3)					
Vaginal bleeding	2 (0.6)	3 (0.3)					
Acute or chronic diseases	7 (2.2)	12 (1.3)					
Late pregnancy	102 (31.8)	338 (35.1)					
Fetal factors	7 (2.2)	12 (1.2)					

*ET* Embryonic transfer, *NT* natural conception

The number of days from admission to birth for treatment to prevent miscarriage was significantly higher in the ET group than in the NC group (*P <* 0.05; See Table 5).

**Table 5:** Comparison of the number of days of miscarriage prevention treatment between two groups

Group	$\overline{x}$ Days of miscarriage prevention (±***s***)	***t***	***P***	***Adjusted OR***	***95%CI***	***P***
Embryo transfer	8.45 ± 7.408	11.145	< 0.001	1.149	1.107–1.193	< 0.001
Natural conception	4.80 ± 4.026					

#### Delivery records

The number of gestational weeks, pregnancy risk classification, mode of delivery, and number of births were significantly different between the ET and NC groups after adjusting for confounding factors (all *P* < 0.05). Compared with the NC group, the ET group had higher percentages of delivery at < 37 weeks, class III risk, cesarean section and twin birth (39.6%). The NC group had comparable proportion of all three classes of risks, a higher proportion of vaginal deliveries, and a higher proportion of single births (98.2%). There were no significant differences in the volume and clarity of amniotic fluid between the two groups (*P* > 0.05; See Table 6).

**Table 6:** Comparison of delivery records between ET and NC groups

Delivery records	ET group, ***n*** (%)	NC group, ***n*** (%)	***χ***^***2***^	***P***	***Adjusted OR***	***95%CI***	***P***
Gestational weeks			17.058	< 0.001	0.659	0.587–0.740	< 0.001
< 37	113 (35.2)	140 (14.5)					
37–40^+ 6^	203 (63.2)	783 (81.3)					
≥ 41 weeks	5 (1.6)	40 (4.2)					
Class of pregnancy risk			492.981	< 0.001	8.969	6.661–12.076	< 0.001
Class I	21 (6.5)	422 (43.8)					
Class II	39 (12.1)	397 (41.2)					
Class III	261 (81.3)	144 (44.9)					
Amniotic fluid volume			2.844	0.092			
Normal	286 (89.1)	822 (85.4)					
Abnormal	35 (10.9)	141 (14.6)					
Amniotic fluid clarity			2.784	0.095			
Clear	285 (88.8)	818 (84.9)					
Not clear	36 (11.2)	145 (15.1)					
Mode of delivery			413.874	< 0.001	3.225	2.656–3.916	< 0.001
Vaginal delivery	65 (20.2)	780 (81.0)					
Cesarean section	251 (78.2)	164 (17.0)					
Vacuum-assisted**/**assisted breech delivery	5 (1.6)	19 (2.0)					
Number of births			345.445	< 0.001	0.023	0.012–0.046	< 0.001
Single	194(60.4)	946 (98.2)					
Twin	127(39.6)	17 (1.8)					

*ET* Embryo transfer, *NC* natural conception

There was no significant difference between the ET and NC groups in the causes of cesarean section (*P* > 0.05; See Table 7).

**Table 7:** Comparison of the causes of cesarean section between ET and NC groups

	Social factors, ***n*** (%)	Pathological factors, ***n*** (%)	***χ***^***2***^	***P***
**ET group**	3 (1.2)	248 (98.8)	0	1
**NC group**	2 (1.2)	162 (98.8)		

*ET* Embryo transfer, *NC* natural conception

### Comparison of neonatal clinical outcomes between embryo transfer and natural conception groups

The percentages for low birth weight and premature birth were significantly higher in the ET group than in the NC group (all *P* < 0.05). In contrast, there were no significant differences in percentages between the two groups for baby gender, fetal macrosomia, fetal anomaly, neonatal asphyxia, extremely low birth weight and umbilical cord conditions (all *P* > 0.05; See Table 8).

**Table 8:** Comparison of neonatal clinical outcomes between ET and NC groups

Neonatal clinical outcomes	ET group, ***n*** (%)	NC group, ***n*** (%)	***χ***^***2***^	***P***	***Adjusted OR***	***95%CI***	***P***
Gender			0.62	0.431			
Male	231 (51.3)	525 (53.6)					
Female	219 (48.7)	455 (46.4)					
Premature birth			24.968	< 0.001	3.267	2.293–4.654	< 0.001
Yes	112 (24.9)	138 (14.1)					
No	338 (75.1)	842 (85.9)					
Fetal macrosomia			0.013	0.911			
Yes	11 (2.4)	23 (2.3)					
No	439 (97.6)	957 (97.7)					
Low birth weight (1.5–2.5 kg)			14.566	< 0.001	3.052	2.110–4.414	< 0.001
Yes	84 (18.7)	110 (11.2)					
No	366 (81.3)	870 (88.8)					
Extremely low birth weight			0	1			
(1.0–1.5 kg)							
Yes	4 (0.9)	8 (0.8)					
No	446 (99.1)	972 (99.2)					
Fetal anomaly			2.577	0.108			
Yes	8 (17.8)	8 (0.8)					
No	442 (98.2)	972 (99.2)					
Neonatal asphyxia			0.031	0.861			
Yes	9 (2.0)	21 (2.1)					
No	441 (98.0)	959 (97.9)					
Umbilical cord conditions			80.454	< 0.001	1.016	0.881–1.172	0.825
Normal	239 (74.5)	651 (67.6)					
Around neck	65 (20.2)	272 (28.2)					
Around foot	1 (0.3)	6 (0.6)					
Twisted	16 (5.0)	34 (3.5)					

*ET* Embryo transfer*, NC* natural conception

## Discussion

In this study, we found that the proportion of geriatric pregnancies was higher in the ET group than in the NC group, as well as the overall age of pregnant women (33.23 ± 4.59 vs 30.16 ± 5.19 years). Geriatric IVF pregnancy was previously reported as having poorer outcomes than IVF pregnancy in younger women. One study found that among women of childbearing age, those aged 20–30 years had the best IVF outcomes and women aged 40 years and older had poor IVF outcomes and a higher rate of miscarriage [4]. As women age, a decline in oocyte production and quality (known as ovarian aging) becomes the primary cause of poorer IVF outcome [5]. We observed that the percentage of women with one pregnancy only was higher in the ET group (48.0%) than in the NC group (31.0%), which was consistent with the findings of Egbe et al. [6] This difference may be attributable to the various causes of infertility in the ET group, meaning women in the ET group were more likely to be having their first pregnancy than those in the NC group. Ectopic pregnancy is the primary cause of early maternal morbidity and mortality, accounting for 1–2% of all pregnancies and ectopic pregnancy incidence has drastically increased with the advancement of ART [7] . The fallopian tube is the most common site of ectopic implantation [8], and about 1.5–2.1% of patients with ectopic pregnancy have undergone IVF [7]. Our findings showed that the percentage of women with a history of ectopic pregnancy was higher in the ET group (15.3%) than in the NC group (2.7%), which may be associated with previous ectopic pregnancy, history of infertility, history of surgery or the use of intrauterine contraceptive device [9].

For gestational comorbidity, the study by Kouhkan et al. [10] showed that women who underwent ET needed more insulin than those in the NC group. Insulin resistance and glucose intolerance during pregnancy can lead to the development of GDM, which explains the higher incidence of GDM in the ET group (42.1%) relative to the NC group (26.4%) in our study.

Preeclampsia is a pregnancy-specific disease with a global prevalence of 5–8%. It is one of the leading causes of maternal and perinatal morbidity and mortality worldwide, causing 50,000 to 60,000 deaths every year [11]. In our study, preeclampsia incidence was higher in the ET group. Preeclampsia is a multisystemic syndrome, and its pathogenesis and pathophysiology involve both genetic and environmental factors [12]. If preeclampsia is not effectively treated in a timely manner, it may endanger the life of the mother and infant or cause sequelae in the short term, and subsequently affect the health of the mother in the long term. Therefore, pregnant women are recommended to complete all prenatal examinations and adhere to a healthy routine and lifestyle. We also found that the incidence of gestational anemia and the number of days of treatment to prevent miscarriage were higher in the ET group (26.0% and 8.45 ± 7.408, respectively) than in the NC group (4.1% and 4.80 ± 4.026, respectively). Most pregnant women who required treatment for miscarriage prevention had threatened labor. The pathogenesis of gestational anemia has been shown to be associated with the age and educational background of pregnant women, as well as a history of ectopic pregnancy [13]. This was consistent with our results, which found that age and incidence of ectopic pregnancy were both higher in the ET group than in the NC group. In addition, ET itself may be a factor associated with gestational anemia. A previous study has demonstrated that severe anemia in pregnant women can lead to premature labor, spontaneous abortion, low birth weight and fetal death [14]. Therefore, implementation of measures to prevent anemia is recommended for women who plan to undergo ET, to ensure maternal and neonatal health.

Analysis of the delivery records revealed that the proportion of women with a high-risk (class III) pregnancy was significantly higher in the ET group (81.3%) than in the NC group (44.9%). Pregnancy risk is primarily classified based on the general conditions of the pregnant woman (age, history of miscarriage, and history of adverse pregnancy), gestational comorbidities (hypertension, anemia, and respiratory infection), and gestational complications (threatened premature labor, GDM, and fetal macrosomia). Age, ART, and twin pregnancy may be the causes for the higher pregnancy risk in the ET group compared with the NC group. The dominant mode of delivery was cesarean section in the ET group (78.2%) and vaginal delivery in the NC group (81.0%). The rate of twin birth was higher in the ET group (39.6%) than in the NC group (1.8%); twin birth is known to be associated with ET. Twin pregnancy imposes certain risks to both maternal and neonatal health, so this issue needs to be carefully considered in the application of ET.

A study by Zhu et al. [15] demonstrated that embryo transfer was associated with higher incidence of premature birth, low birth weight and small-for-gestational age infants. Here, we found that premature birth and low birth weight were observed in 38.8 and 36.5% of women with ET, respectively. The risk of premature birth is higher among women undergoing IVF, but such risk is mostly believed to be a secondary consequence of the significant increase in multiple pregnancies [16]. Our study also showed that the ET group had a higher incidence of twin pregnancy. Furthermore, Qin et al. [17] demonstrated that low birth weight was positively correlated with ART, which was consistent with our findings that the proportions of premature and low birth weight babies were higher in the ET group (24.9 and 18.7%, respectively) than in the NC group (14.1 and 11.2%, respectively) when we compared neonatal clinical outcomes. After the correction of confounding factors, compared with NC group, ET group had no statistical significance in umbilical cord condition, but the abnormal condition of umbilical cord should be paid attention to. The umbilical cord is a conduit between the fetus and the placenta that mediates substance exchange between the fetus and mother. Umbilical cord complications are generally considered to be the root cause for chronic intrauterine hypoxia, reduced fetal movement, growth retardation and oligohydramnios [18]. Therefore, regular prenatal examinations are recommended for pregnant women, especially ultrasound examination during the second and third trimesters of pregnancy, to ensure early identification of umbilical cord abnormality.

In summary, the ET group had poorer maternal clinical outcomes than the NC group and higher incidences of premature birth and low birth weight. These differences may be associated with maternal physical fitness, nutritional status, financial status, past health status, ET, and higher rates of reported adverse outcomes due to closer monitoring of pregnant women with ET. Women who plan to have ET are recommended to undergo the procedure at an appropriate reproductive age, maintain physical fitness and good nutrition, and take good prenatal care.

## Conclusions

The clinical outcomes of mothers and the birth status of infants were better in the NC group than in the ET group. Maternal health must be closely monitored and improved in the ET group to reduce the incidence of gestational comorbidity and enhance the quality of fetal life.

## Data Availability

The datasets used and/or analyzed during the current study are available from the corresponding author on reasonable request.

## Abbreviations

ET: embryo transfer
NC: natural conception
IVF: in vitro fertilization
ART: assisted reproductive technology
IBM: International Business Machines Corporation
GDM: gestational diabetes mellitus
